# Progress of Obstetrics

**Published:** 1875-02

**Authors:** E. Warren Sawyer

**Affiliations:** Lecturer on Obstetrics, Rush Medical College, Chicago


ARTICLE II.

### PROGRESS OF OBSTETRICS.

By E. WARREN SAWYER, M.D.,

LECTURER ON OBSTETRICS, RUSH MEDICAL COLLEGE, CHICAGO.

1. How do the Spermatozoa enter the Uterus? By JOSEPH R. BECK, M.D., Fort Wayne, Indiana. (*American Journal of Obstetrics and Diseases of Women and Children.* New York, November, 1874. Read before the American Medical Association, June 2, 1874.

1. In the above paper, which has the first place in the last number of the Journal of Obstetrics, the author has made a prolonged and wordy attempt to answer the question which apparently has concerned him not a little; and which, he informs us, has never before been answered. How far his effort has placed this question beyond doubt, the reflective reader will decide.

The first dozen pages are devoted to a tirade against all previous observers, for no other reason, it seems to us, than because they failed years ago to see what he has recently seen. We are surprised at this disposition, because it occurs to us as better taste to respect our teachers for what we have learned of them, and not to censure them for what they could not teach.

We are not conscious of a real need of apologizing for the omissions of those whose works have already made them known; but when he concludes that the doubts and opinions of Gardner place this observer "on the top of the fence," we ask permission of the author to remind him that it is always honest and often prudent to doubt, when we are not convinced; and if Gardner is really on the top of the fence in this matter, perhaps it is because that aspirating womb, in Fort Wayne, has not yet drawn him to either side.

Of Byford's work on the uterus, which he has vainly consulted on this point, he says "we might have expected some information, no matter how erroneous." We sincerely hope that this conclusion of the author is not the expression of a principle which guides him in imparting knowledge to others ; but rather that he seeks to have his observations confirmed, at least before they are given to the world as unquestionable truths.

Thomas escapes comment by the author because, he says, "there is nothing to comment upon ;" though in the next edition of Thomas' work he hopes there will be more.

He is loud in praise of Sims ; who "*almost came upon the truth.*" It seems to us that the theories of Sims detract from the originality of the author.

On page 363, we are startled with the definition of spermatozoids ; (he prefers to call them spermatozoa,) "they are simply ciliated cells." If, indeed, the author has discovered that they are ciliated cells, it is not impossible that in this fact is the explanation of their peregrinations ; may they not then be capable of extensive wanderings, even from the testicle to the ovary ?

At last the author asserts that he will "answer the question in a positive manner, by a description of what he saw." Then follows an account of a married lady ; a blonde of strongly marked nervous temperament ; who came to be treated by him for a falling of the womb, which she had had since her abortion, six years before. Being cautioned by the lady to conduct his examination with great care, as her orgasm was easily aroused because of her passionate nature, he concludes that this is an opportunity for experimentation, such as had never before been offered to any one, and which should not now be lost. He now offends good taste by detailing how, when the mouth of the womb was brought clearly into view in the sunlight, he swept the right forefinger quickly three or four times across the space between the cervix and pubic arch, when almost instantly the orgasm occurred." Now mark what he saw ; the mouth of the womb open to the extent of fully an inch ; make five or six successive gasps, and instantly all was as before. What a spectacle ! Fortunately for the lady, and perhaps for science, the gasping womb did not seize upon the observer at this moment.

We are clearly told that the aspiration, which the uterus exerts is the explanation of the passage of the spermatozoids "*to their destination.*"*

This aspirating force of the uterus can only be comprehended by recalling its alleged capacity of drawing the spermatozoids to their destination, which physiologists have agreed is some part of the Fallopian tube. Engineers may learn something from this ; for here are overcome greater physical obstacles than are presented in the scheme of a pneumatic railway.

The temerity of husbands would be something remarkable, did they continue their marital relations after being informed of this snapping-turtle function of a woman's uterus during coition.

It is but fair to say, that the author liberally admits, in exceptional instances, some other way of the passage of the sperm ; but his is the usual way.

With corresponding liberality, we are ready to admit that what he saw, may explain the entrance of sperm into *that* womb, but we do not admit that the opening of the mouth of the womb, and the suction force of the organ, *are common phenomena*, or that they are necessary to the entrance of the sperm into the uterine cavity. We conclude this for four reasons, viz. :

*First.* Because impregnation has been known, more than once, to follow the depositing of semen upon the external genitalia, far out of the reach of the womb.

*Second.* There are some women who slip a tightly fitting rubber cap over the cervix uteri during coition ; by which the cavity of the uterus is effectually closed. Now, how could any such remarkable opening and enlargement of the neck take place, during the orgasm, without painfully constricting the neck in the unyielding ring of the so-called "preventive pessary"; or with-

* Reviewer's italics.

out breaking this latter? We have never known either accident to occur. On the other hand, *we have known those women to become pregnant, who had sealed their womb against the entrance of the sperm, but who had incautiously removed this seal too soon after sexual intercourse, though hours after the orgasm had subsided.* It is now a common conclusion, among those women, that there is no security against impregnation, by wearing the pessary alone, *if, beside, the semen is not washed out of the vagina soon after coition.*

*Third.* Such a function of the uterus is incompatible with the state of pregnancy; for, should this sudden and remarkable successive opening and closing of the organ occur, while it contained an ovum, the latter would be most surely detached and expelled; in other words, the woman would abort the first time her orgasm was aroused; such, however, is not the case.

*Finally.* We do not think the opening and gasping of the uterus are constant freaks of the organ, because Dr. Wernich, from whom the author quotes, did not observe them; and his opportunities for observation were even fairer then the author's, because the position of all the parts was normal.

The last third of this paper is allowed to an interesting and profitable article "On the erectile properties of the lower segment of the uterus and its significance," by Dr. Wernich; who hopes some day to prove that a certain mechanism of the cervix uteri, which the erection induces, is necessary to conception.

We also conclude this review, hoping soon to be possessed of more well founded facts, connected with this interesting physiological problem.

